# Resident Attitudes Toward Performing Pelvic Examinations in the Emergency Department

**DOI:** 10.1089/whr.2022.0084

**Published:** 2023-02-17

**Authors:** Isha Agarwal, Deesha Sarma, Ryan C. Burke, Matthew Babineau, Scarlet Benson, Tania Strout, Leslie A. Bilello, Leon D. Sanchez

**Affiliations:** ^1^ Department of Emergency Medicine, Maine Medical Center, Portland, Maine, USA.; ^2^ Department of Emergency Medicine, Beth Israel Deaconess Medical Center, Boston, Massachusetts, USA.; ^3^ Department of Emergency Medicine, Dartmouth-Hitchcock Medical Center, Lebanon, New Hampshire, USA.; ^4^ Department of Emergency Medicine, Aventura Hospital and Medical Center, Aventura, Florida, USA.; ^5^ Department of Emergency Medicine, Brigham and Women's Faulkner Hospital, Boston, Massachusetts, USA.

**Keywords:** gynecology, health disparities, pelvic examination, resident education

## Abstract

Mounting evidence suggests that emergency physicians tend to avoid patients with gynecologic chief complaints, and that avoidance may be higher for male physicians compared to females. One underlying reason could be discomfort with performing pelvic examinations. The goal of this study was to assess whether male residents report greater discomfort with pelvic examinations than females. We performed a cross-sectional, Institutional Review Board-approved survey of residents at 6 academic emergency medicine programs. Of 100 residents who completed the survey, 63 self-identified as male, 36 female, and one selected “prefer not to say” and was excluded. Responses were compared between male and females using chi-square tests. In secondary analysis, *t*-tests were used to compare preferences for various chief complaints. Self-reported comfort with pelvic examinations did not differ significantly between males and females (*p* = 0.4249). Barriers for male respondents in performing pelvic examinations included lack of training, general dislike, and concern the patient would prefer female providers. Male residents had a statistically significant higher aversion ranking towards patients with vaginal bleeding than female residents (mean difference = 0.48, confidence interval = 0.11–0.87). Aversion ranking was the same between males and females on other chief complaints. There is a gender disparity among male and female residents in attitudes towards patients with vaginal bleeding. However, the results from this study do not demonstrate a significant difference in self-reported comfort amongst male and female residents in performing pelvic examinations. This disparity may be driven by other barriers, including self-reported lack of training and concern about patients' physician gender preferences.

## Introduction

Although gynecologic complaints represent a substantial proportion of emergency department (ED) visits,^1^ prior studies have shown that emergency physicians tend to avoid these patients.^2^ In one study of all ED chief complaints, patient pickup time was longest for vaginal bleeding (12 minutes) compared to a median of 6 minutes for all other chief complaints.^2^ Differences in patient avoidance may be driven by inherent physician characteristics. A recent study found that male residents were less likely than female residents to assign themselves to patients with a chief complaint of vaginal bleeding.^3^

One deterrent for seeing patients with gynecologic chief complaints may be that the workup requires performing a pelvic examination.^4^ Performing pelvic examinations can be time-intensive and detract from ED throughput.^5^ They are also labor and resource-intensive, requiring examination rooms offering privacy and a chaperone present for the duration of the examination.^6^ Although basic procedural training is an integral aspect of medical education, there is a wide range of experience and comfort with procedures among incoming resident physicians.^7^ The goal of this study was to investigate self-reported resident physician comfort with performing pelvic examinations. In particular, we hypothesized that male residents may report greater discomfort with performing pelvic examinations than female residents. We aimed to better understand key barriers to performing pelvic examinations in the ED, to highlight opportunities for facilitating this procedure and improving resident education and training.

## Methods

### Survey design and recruitment

We performed a cross-sectional, Institutional Review Board (IRB)-approved survey of emergency medicine (EM) residents at six academic EM programs across the United States. These programs were chosen as part of a convenience sample based on known contacts of the study research team at other academic institutions. The Principal Investigator emailed the program directors and/or program coordinators of these institutions to assess their willingness to participate. Those who opted in sent a recruitment invitation email to their residents containing the survey link.

The survey was administered through the Research Electronic Data Capture (REDCap) database, a secure, web-based application, allowing for deidentified links to collect anonymous data. A maximum of 3 survey email reminders were sent. No identifiable data were collected and data were analyzed in aggregate. The respondents did not receive any compensation nor reimbursement for participation. The survey questions were adapted from a prior, internal IRB-approved study investigating resident self-assignment to patients with various chief complaints. The survey was then internally tested and modified by three EM physicians within the research team before being circulated.

### Survey questions

The primary measure was self-reported comfort with performing pelvic examinations, defined as either bimanual or speculum examination. Secondary measures included self-reported preferences for various chief complaints (vaginal bleeding, epistaxis, gastrointestinal [GI] bleeding, and laceration), which were chosen because they require either an invasive medical examination or procedure as part of the workup. Respondents selected from 1 to 5 on a preference scale where 1 represented “strong preference” and 5 represented “strong aversion.”

Respondents were asked the number of pelvic examinations they performed over the span of five shifts and whether they believe they perform pelvic examinations more, less, or the same on average compared to their peers. They were also asked about training received in performing pelvic examinations, whether an obstetrics and gynecology (OBGYN) rotation in medical school or residency, a simulation on a task trainer, mannequin, or standardized human patient, or supervised pelvic examinations in the ED. Finally, respondents were asked to report barriers to performing pelvic examinations.

### Survey analysis

Data were summarized overall and by gender. Comparisons between male and female residents were done with *t*-tests for continuous variables and chi-square tests for categorical variables. All analyses were performed in SAS version 9.4.

## Results

Of the 209 residents who were recruited for participation, 100 responded (48% response rate). Of the respondents, 63 self-identified as male, 36 as female, and 1 selected “Other/Prefer not to say” and was excluded from the gender-based comparative analysis. The majority (78%) of respondents were in 3-year residency programs, while 22% were in 4-year programs. Respondents were split across postgraduate year (PGY), with 31% in PGY1, 28% in PGY2, 36% in PGY3, and 5% in PGY4. In terms of geographic spread, 42% of respondents were in programs located in New England, 20% in the Southeast, and 38% in the Midwest or West.

**FIG. 1.**
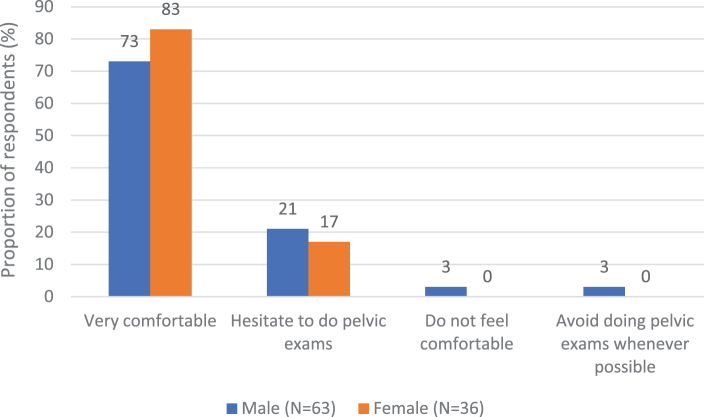
Self-reported personal comfort with performing pelvic examinations by gender.

For the primary measure, we found that self-reported comfort with pelvic examinations did not differ significantly between males and females (*p* = 0.4249), with 83% of female respondents reporting feeling “Very comfortable” with performing pelvic examinations, compared to 73% of male respondents (Fig. 1).

**FIG. 2.**
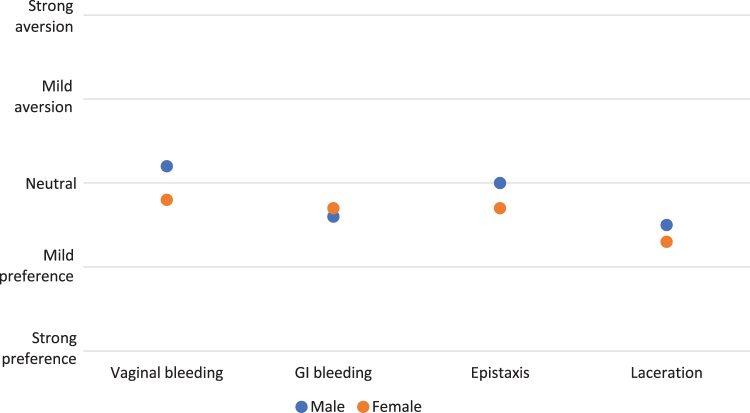
Self-reported preference for seeing patients with chief complaint by gender. GI, gastrointestinal.

Male residents had a statistically significant higher aversion ranking for patients with a chief complaint of vaginal bleeding compared to female residents (mean difference = 0.48, confidence interval [CI] = 0.11–0.87). Mean preference ranking was not significantly different between males and females on the other chief complaints of GI bleeding, epistaxis, or laceration (Fig. 2).

Females reported a slightly higher, although not statistically significant, number of pelvic examinations performed over a span of five shifts (mean = 2.1, CI = 1.6–2.5) than males (mean = 1.7, CI = 1.3–2.0).

Respondents were able to select any number of applicable barriers to performing pelvic examinations. Self-reported barriers for males included difficulty finding a chaperone (41%), concern that the patient will want a female provider (41%), general dislike (33%), and lack of training (20%). Although male residents reported a lack of training, 67% (CI = 54%–78%) of male residents reported supervised pelvic examinations with faculty in the ED as part of their training, compared to 36% (CI = 21%–54%) of female residents.

The most common self-reported barriers for female respondents included concern that the patient would be physically uncomfortable during the procedure (38%) and difficulty finding a private space for the examination (36%), and difficulty finding a chaperone (22%).

## Discussion

Our study found that there is a gender disparity among male and female EM residents in attitudes toward patients with vaginal bleeding, with a significantly higher aversion ranking for this chief complaint for male residents compared to female residents.

However, the results from this study do not demonstrate a statistically significant difference in self-reported comfort among male and female residents in performing pelvic examinations. This discrepancy indicates that there are influences apart from physician comfort that led to a lower preference ranking for male residents toward patients with a chief complaint of vaginal bleeding. One factor may be the concern that patients with vaginal bleeding prefer a female provider; this was the most common barrier to performing pelvic examinations that male residents reported in our survey. To this point, prior studies have indicated that a substantial number of women who received care in academic training sites refuse to be examined by a male resident.^8^

One study found that women who did not know the training level of the resident were more likely to refuse, and many female patients listed reasons such as “I am more comfortable with a female,” and “Women explain things better.”^8^ Another barrier male residents reported with respect to performing pelvic examinations is a lack of training. However, most male residents reported already participating in supervised pelvic examinations with ED faculty as part of their training. Therefore, adding more structured educational initiatives to residency training could be beneficial. A simulation curriculum could be implemented during residency orientation or intermittently into weekly didactics, to ensure uniform training and minimize any gender-based disparities in providing gynecologic care.

### Limitations

There were several limitations to this study. This was a pilot study and as such had a small convenience sample of EM residents. In addition, there was a lower response rate of around 50%. Despite this, there was representation across multiple geographic regions, which may improve generalizability. However, we did not have sufficient power to analyze differences in experience or comfort with pelvic examinations by PGY and gender. Self-reporting bias may have influenced responses, biasing results toward the null. In addition, the nonspecific definition of a pelvic examination used in the survey may not capture potential differences between speculum examinations and bimanual examinations.

## Conclusions

Our results suggest that male residents are more averse than female residents to performing pelvic examinations in the ED. Although we did not detect a difference in self-reported comfort with performing pelvic examinations or number of pelvic examinations performed, these findings are limited by their self-reported nature. Nevertheless, the results from this study support further work to better understand the educational, social, and institutional factors that lead to sex-based disparities in attitudes toward patients who present to the ED with gynecologic chief complaints such as vaginal bleeding.

## Abbreviations Used

CI: confidence interval
ED: emergency department
EM: emergency medicine
GI: gastrointestinal
IRB: Institutional Review Board
PGY: postgraduate year

## References

[1] ACOG Practice Bulletin: Clinical Management Guidelines for Obstetrician-Gynecologists. Committee on Practice Bulletins-Gynecology, 2006. Available from: https://pubmed.ncbi.nlm.nih.gov/16648432/ [Last accessed: August 5, 2021].

[2] Patterson BW, Batt RJ, Wilbanks MD, et al. Cherry picking patients: examining the interval between patient rooming and resident self-assignment. Acad Emerg Med 2016;23(6):679–684. 26874338 10.1111/acem.12895

[3] Agarwal I, Grossestreuer A, Joseph JW, et al. Association of resident characteristics with patterns of patient self-assignment. Am J Emerg Ed 2021;44:112–115. 10.1016/j.ajem.2021.01.081 33588250

[4] McLean ME, Santiago-Rosado L. Plight of the pelvic examination. Em Med J 2019;36(6):383. 10.1136/emermed-2019-208474 30996029

[5] Radecki R. Excitement and Ennui in the ED. Available from: https://www.emlitofnote.com/?p=3132 [Last accessed: August 5, 2021].

[6] Brown J, Fleming R, Aristzabel J, et al. Does pelvic examination in the emergency department add useful information? West J EM 2010;12(2):208–212. PMC3099609 21691528

[7] Murdoch W, Porcerelli J, Markova T, et al. Incoming resident experience and comfort with procedures designated as “basic.” Fam Med 2012;44(1):47–50. 22241341

[8] Rifkin JI, Shapiro H, Regensteiner JG, et al. Why do some women refuse to allow male residents to perform pelvic examinations? Acad Med 2002;77(10):1034–1038. 12377683 10.1097/00001888-200210000-00020

